# Interspecific studies of circadian genes *period* and *timeless* in Drosophila

**DOI:** 10.1016/j.gene.2018.01.020

**Published:** 2018-03-30

**Authors:** Shumaila Noreen, Mirko Pegoraro, Faisal Nouroz, Eran Tauber, Charalambos P. Kyriacou

**Affiliations:** a Department of Genetics and Genome Biology, University of Leicester, United Kingdom; b Molecular Genetics Lab, Department of Zoology, University of Peshawar, Pakistan; c Department of Evolutionary & Environmental Biology, The Faculty of Natural Sciences, University of Haifa, Haifa 3498838, Israel

**Keywords:** bp, base pair, CLK, clock, CRY, cryptochrome, CYC, cycle, D., Drosophila, *D. mel*, *D. melanogaster*, *D. ps*, *D. pseudoobscura*, DAM, *Drosophila* activity monitoring, DD, constant darkness, DD, continuous dark, DNA, deoxyribonucleic acid, E, evening, EP, evening peak, g, grams, h, hour, H, hour, L, litre, LD, light dark cycle, M, morning, min, minute, MP, morning peak, n.a., not available, ns, not significant, PAS, Per Ant Sim, PCR, polymerase chain reaction, *per*, period, Pers comm, personal communication, RNA, ribonucleic acid, SD, standard deviation, sec, second, SEM, standard error of the mean, *tim*, timeless, TTFL, transcriptional translational feedback loop, ZT, Zeitgeber time, Drosophila, Circadian rhythms, Period, Timeless, Coevolution, *D. pseudoobscura*

## Abstract

The level of rescue of clock function in genetically arrhythmic *Drosophila melanogaster* hosts using interspecific clock gene transformation was used to study the putative intermolecular coevolution between interacting clock proteins. Among them PER and TIM are the two important negative regulators of the circadian clock feedback loop. We transformed either the *D. pseudoobscura* per or tim transgenes into the corresponding arrhythmic *D. melanogaster* mutant (per01 or tim01) and observed >50% rhythmicity but the period of activity rhythm was either longer (*D. pseudoobscura*-per) or shorter than 24 h (*D. pseudoobscura*-tim) compared to controls. By introducing both transgenes simultaneously into double mutants, we observed that the period of the activity rhythm was rescued by the pair of hemizygous transgenes (~24 h). These flies also showed a more optimal level of temperature compensation for the period. Under LD 12:12 these flies have a *D. pseudoobscura* like activity profile with the absence of morning anticipation as well as a very prominent earlier evening peak of activity rhythm. These observation are consistent with the view that TIM and PER form a heterospecific coevolved module at least for the circadian period of activity rhythms. However the strength of rhythmicity was reduced by having both transgenes present, so while evidence for a coevolution between PER and TIM is observed for some characters it is not for others.

## Highlights

• To understand the mechanism of intermolecular coevolution between *period* and its partner *timeless*, transgenes from *D. pseudoobscura* were introduced into *D. melanogaster* null mutants.
• The single *D. melanogaster* transformants containing either *per* or *tim* from *D. pseudoobscura* displayed less than 50% rhythmicity and the period of locomotor activity rhythms was also either very long (26-31h for *per*^*ps*^) or very short (21-22h for *tim*^*ps*^).
• The two heterospecific transgenes together in the double mutant *D. melanogaster* flies could not show a significant increase in levels of rhythmicity however a significant improvement in the average period of locomotor activity was obtained (~24h).
• Temperature compensation in the period of locomotor activity was also improved in these double tranformants. The Western blots and qPCR confirmed the expression of these transgenes.

## 1 Introduction

The molecular basis of the circadian clock has been extensively studied in several model species and has led to the idea that the general mechanism that underlies the clock is conserved. It consists of interlocked auto-regulatory feedback loops that function through the transcription/translation of positive and negative elements (Bell-Pedersen et al., 2005). In *Drosophila* the basic model for rhythm generation in the pacemaker cells involves several core genes, *period* (*per*), *timeless* (*tim*), *Clock* (*Clk*), *cycle* (*cyc*) and *cryptochrome* (*cry*). The interaction of the protein products of these genes with associated kinases and phosphatases leads to pace-setting of the clock by regulating the timing of nuclear entry and inter-molecular interactions (Peschel and Helfrich-Förster, 2011; Zheng and Sehgal, 2008).

*D. melanogaster* has been transformed with different species clock genes and the level of rescue of arrhythmic null mutants has been used as a tool to study interspecific functional conservation and species-specific characters (Petersen et al., 1988; Wheeler et al., n.d.; Peixoto et al., 1998; Tauber et al., 2003). PER and TIM are the two key clock proteins that mediate the negative limb of the circadian feedback loop. TIM binds to the PAS domain of PER (Hardin, 2011) and prevents its degradation (Kloss et al., 1998). Phylogenetic analysis of *tim* from *D. virilis* and *D. hydei* revealed that TIM, is more conserved than PER (Ousley et al., 1998). Ousley et al. (1998) also reported the first robust rescue of the *tim*^*01*^ mutant using a conspecific *tim* transgene and Nishinokubi et al. (2006) showed that the *D. ananassae*-*tim* transgene was also able to rescue behavioural rhythms of *D. melanogaster tim*^*01*^ mutants. In another study the same group induced the *D. ananassae* TIM protein in *D. melanogaster tim*^*01*^ transformants through heat shock and examined the behaviour of these flies (Nishinokubi et al., 2006). The level of TIM protein was increased initially by the application of heat shock and decreased after some time. Their results demonstrated that by applying this heat shock at different times of the day, these transgenic *D. melanogaster tim*^*01*^ flies became nocturnal, similar to wild-type *D. ananassae*. They also found that mating activity rhythms of the transformants were different from both parental species, suggesting that they are determined by different factors from those which control locomotor activity rhythms. Nevertheless, their results suggested that like *per* (Wheeler et al., n.d.; Tauber et al., 2003), *tim* might also play a role as a speciation gene and control some aspects of adaptive rhythmic behaviour.

While the transformation of interspecific clock genes into *D. melanogaster* hosts provides information on species-specific characteristics, the experimental paradigm usually involves introducing a single interspecific transgene. In one of these studies, phylogenetic analysis of the PER PAS interaction domain was correlated with the efficiency of *per*^*01*^ rescue (Piccin et al., 2000). Specifically, the *Musca domestica per* transgene gave better rescue that the *D. pseudoobscura* transgene even though *Musca* had a common ancestor with *D. melanogaster* much earlier than *D. pseudoobscura*, yet the phylogeny of the PER-PAS domain revealed that *Musca* lay closer to *D. melanogaster* than *D. pseudoobscura* (Piccin et al., 2000). As PER-PAS dimerises with TIM, these results may represent a coevolution of TIM with PER which might be reflected at the phylogenetic level in TIM sequences and functionally in rescue experiments. Consequently we asked whether the relatively poor rescue of *D. melanogaster per*^*01*^ rhythmicity by the *D. pseudoobscura per* transgene, which is about 50% but with longer periods of 27–28 h (Petersen et al., 1988; Peixoto et al., 1998), might be enhanced by performing a double interspecific transformation using *per* and *tim* transgenes from this species. Under a coevolution scenario we might expect an improved rescue if the PER-TIM interaction coevolves as a separate unit. However, introducing a second interspecific transgene could actually make rescue worse if the coevolving unit includes the positive regulators CLOCK and CYCLE, which also physically interact with PER-TIM (Lee et al., 1996).

Consequently, we co-inserted the *D. pseudoobscura per* and *tim* transgenes into the corresponding double mutant background *per*^*01*^; *tim*^*01*^ to test this coevolution scenario. In addition we also investigated whether any switching of *D. melanogaster* host circadian behavior to that of *D. pseudoobscura* occurred, as reported in *D. pseudoobscura-per* transformants for both species-specific locomotor and mating rhythms (Petersen et al., 1988; Tauber et al., 2003).

## 2 Materials and methods

### 2.1 The transgenic lines

The following *D. melanogaster* mutant lines were used *2A*; *per*^*01*^ transformant strain carrying the 13.2 Kb *D. melanogaster per* transcription unit *per*^*mel*^, (Citri et al., 1987) *I26*; *per*^*01*^ transformant strain carrying the *D. pseudoobscura per* coding sequence (*per*^*ps*^) fused to the upstream non-coding *melanogaster per* sequences (Petersen et al., 1988).

*t28s*; *tim*^*01*^ transformant strain carrying the *D. melanogaster tim* transcription unit (*tim*^*mel*^).

*tim19*, *tim21*, *tim35*; *tim*^*01*^ transformant strains carrying the *D. pseudoobscura tim* (*tim*^*ps*^) transcription unit with full length *pseudoobscura* TIM coding sequence attached to the *melanogaster tim* promoter. All of them were marked with *w+*.

**Table 1:** Primers used for the genotyping of the transgenic flies.

No.	Primer name	Primer sequence	Annealing temperature
1.	*DmTim0 F*	*GCTCATCGCTTTTCATATGTT*	57
2.	*DmTim R*	*AGGATGTGATTGGTAACCAC*	57
3.	*DmPer0 F*	*TACCACCACGAGGACCTCTC*	57
4.	*DmPer R*	*GATGGTGTCCGACGACAAAT*	57
5.	*Pseudoobscura per F*	*ACCACCACGATGACCTCCCC*	59
6.	*Pseudoobscura per R*	*TTGTTCTGCAACTCCTCCGCG*	59
7.	*Pseudoobscura tim F*	*ACATACCGGAAACGCACGGG*	59
8.	*Pseudoobscura tim R*	*CTTGTAGATCAGCGCGATCAAC*	59

These lines were genotyped using PCR for the presence of *tim*^*01*^, *tim*^*+*^, *per*^*+*^, *per*^*01*^ and also for the transgenes of *tim*^*ps*^ and *per*^*ps*^ (Table 1). The location of the *per*^*ps*^ inserts was already known from Peixoto et al. (1998). Male flies carrying the *tim*^*ps*^ transgenes, were crossed to double autosome balancer virgin females *w*; *CyO/Sco*; *TM6b/MKRS* to map the inserts, all of which were located on chromosome II. The transgenes; *per*^*ps*^ and *tim*^*ps*^ were brought together following a series of crosses (see Supplementary Fig. 1) to obtain the homozygotes *per*^*01*^; *tim*^*01*^, *tim*^*ps*^; *per*^*ps*^.

### 2.2 Phylogenetic analysis

To infer the phylogenetic tree the protein sequences of PER, TIM, CLK, CYC and CRY of 12 *Drosophila* species (*melanogaster*, *pseudoobscura*, *sechellia*, *virilis*, *simulans*, *yakuba*, *ananassae*, *willistoni*, *persimilis*, *erecta*, *mojavensis*, *grimshawi*) were downloaded in FASTA format from FlyBase database (http://flybase.org/blast/checkJobStatus.html). Due to unavailability of complete *Clock* gene sequences in FlyBase, the sequences were collected from NCBI (https://blast.ncbi.nlm.nih.gov/Blast.cgi). To filter out the false positives, the resultant hits with >70%query coverage and identity were collected and analysed. To construct the phylogenetic tree, the downloaded sequences from the 5 genes were aligned in CLUSTALW multiple sequence alignments implemented in BioEdit (Hall, 1999). The nucleotide polymorphisms and variations were investigated in BioEdit. The phylogenetic analysis was performed by constructing the un-rooted Neighbor-Joining tree with 1000 bootstrap replicates implemented in MEGA5 (Tamura et al., 2011). The p-distance method was used to compute the evolutionary distances.

### 2.3 Behavioural analysis

Circadian locomotor activity of flies was recorded in Trikinetics monitors (Waltham, Ma, USA). Individual male flies were loaded into glass tubes containing sugar food. One end of the tube was closed with a cap and other end with cotton plug. Behavioural analysis was performed for all strain both at 25 °C and 18 °C. Initially the flies were kept for three to four days in 12:12 light/dark cycles (LD12:12) and in (constant darkness) DD for a further 7–10 days. The activity events were arranged into 30 min bins and CLEAN, a high resolution spectral analysis was used to obtain the free-running DD period, in addition to autocorrelation and actograms of individual flies (Rosato and Kyriacou, 2006). The average genotype locomotor activity profiles over 24 h were constructed in excel by using “Befly!” software (Allebrandt et al., 2013).Statistical analysis was performed using Statistics 5 and Oriana programs (Pegoraro et al., 2014). The activity profiles of the transgenic flies were compared at different temperatures as well as in comparison to each other for the morning (M) and evening (E) components of activity. Bins for those particular phases of activity were highlighted and statistical analysis was performed using the circular statistics package “Oriana” which implements the Watson-Williams F-Test.

### 2.4 Western blots

Western blots were performed for PER proteins using polyclonal anti-PER (a gift from Ralf Stanewsky). Flies were entrained under LD 12:12 on either 25 °C or 18 °C and collected in liquid nitrogen at different time intervals. Protein was extracted from the heads of about 50 flies. Polyacrylamide gels were prepared and proteins were loaded as per the protocol described in Pegoraro et al. (2014).

### 2.5 Quantitative real-time PCR

qPCR was performed to check the expression of *tim*. Flies were entrained under LD 12:12 at 25 °C for 3 days and were collected in liquid nitrogen at ZT12. RNA was extracted from the heads and qPCR by using trizol. The following primer pair FTIMpseud1 (*GATCTGCTGGGATGGACGAT*) and RTIMpseud1 (*GCCACCTCGTTGTCACACTC*) was used for the amplification of cDNA. They were designed against the variable regions between the two species genes.

## 3 Results

### 3.1 Protein sequence analysis

**Fig. 1:**
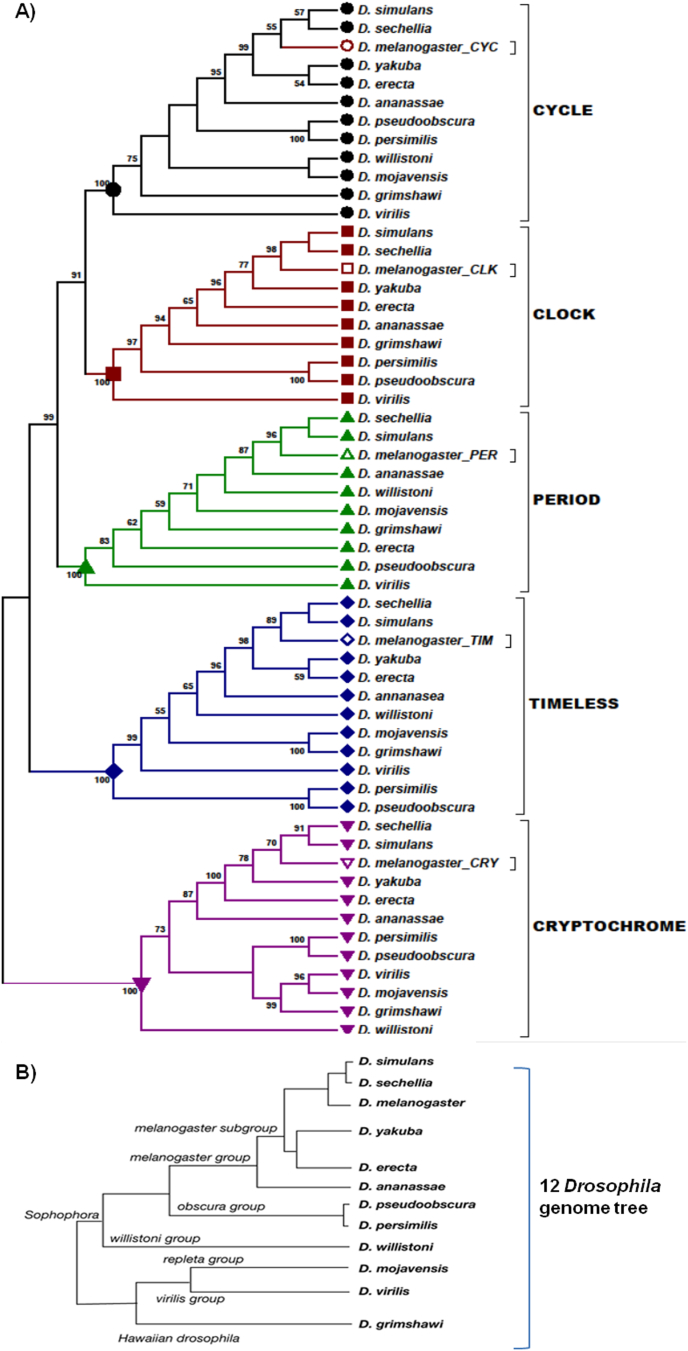
Phylogenetic analysis of circadian genes in 12 *Drosophila* species. The tree was generated by Neighbor-Joining method in Mega5. The p-distance model was used to calculate the genetic distance. Numbers at the bases of branches refer to bootstrap values (%). B) Reference phylogram from 12 *Drosophila* genome project created using pair wise genomic mutation distances.

The protein sequences encoded by the clock genes *per*, *tim*, *Clock*, *cyc* and *cry* were compared using the genomes from the 12 *Drosophila* species (Clark et al., 2007) particularly focusing on *D. melanogaster* and *D. pseudoobscura*. Protein sequences of TIM were aligned and the level of similarity obtained (Fig. 1). The similarity between *D. melanogaster* and *D. pseudoobscura* TIM was 75%. The *D. pseudoobscura/persimilis* group was clustered further away from *D. melanogaster* than all other *Drosophila* species investigated. For PER, the position of *D. pseudoobscura* was further from other *Drosophila* species except *D. virilis* (Fig. 1).

For CLK the position of *D. pseudoobscura* was further from *D. melanogaster* than *D. ananassae* and *D. grimshawi*, while *D. virilis* was the most distant species. The phylogenetic analysis of CYC showed *D. pseudoobscura* closer to *D. melanogaster* than *D. mojavensis*, *D. grimshawi* and *D. virilis*. The CYC protein sequence similarity was 70% between *D. melanogaster* and *D. pseudoobscura*. The sequence similarity for CRY was 81% between *D. melanogaster* and *D. pseudoobscura* but the topology of the tree is rather different from those of the previous clock proteins. In particular, *D. pseudoobscura* and *D. persimilis* CRY is relatively closer to *D. melanogaster* than *D. virilis* and *D. willistoni* (Fig. 1a). Of all the trees, this one resonates with the accepted phylogenetic positions in the 12 *Drosophila* genome project (Fig. 1b). Considering the evolutionary distance between all *Drosophila* species, the position of the *D. pseudoobscura/persimilis* clade is anomalous for PER, CLK and TIM but not for CRY and CYC.

### 3.2 Locomotor activity rhythms

Behavioural analysis was performed on all the transgenic (hemizygous and homozygous) and control lines at 25 °C and 18 °C. Flies were entrained under LD12:12 for 3–4 days and then placed in DD for 7–10 days.

**Fig. 2:**
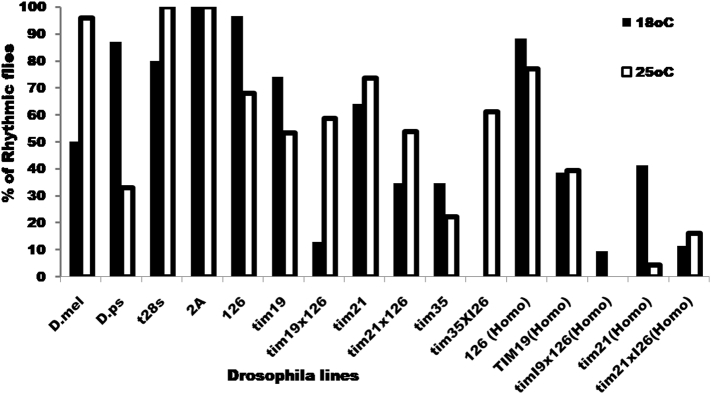
Locomotor rhythms detected in hemizygous and homozygous transgenic flies at 18 and 25 °C and percent rhythmic flies innes under 18 and experimental and control lines.

The parental *D. melanogaster* and *D. pseudoobscura* strains showed interesting temperature-dependent circadian phenotypes, with *D. pseudoobscura* showing higher levels of rhythmicity at lower temperatures (18 °C), with *D. melanogaster* showing the opposite phenotype (Fig. 2, Table 2). This species-specific phenotype was partially reflected in the corresponding transformants *D. melanogaster* lines carrying *tim*^*mel*^ (*melt28s*) which generated higher levels of rhythmicity at 25 °C whereas *per*^*mel*^ (*mel2A*) showed 100% rhythmicity at both temperatures. Similarly, the *D. melanogaster per*^*ps*^ transformant lines *I26* and *tim19* revealed much higher levels of rhythmicity at the colder temperatures (Fig. 2, Table 2) whereas *D. melanogaster tim*^*ps*^ line *tim21* revealed a similarly good rescue at both temperatures. However, when the *tim*^*ps*^ transgenes were co-expressed with the *per*^*ps*^ (*I26*) transgene, a similar level of rescue was observed compared to the single transgenes at 25 °C but this was significantly reduced at lower temperatures. One other hand *D. melanogaster per-tim* double transformant (from the cross *I26* × *tim35*) generated low levels of rescue at both temperatures (Table 2).

**Table 2:** Results of the activity analysis for the hemizygous transgenic and control flies used in this study under 25 °C and 18 °C. Chi-square test was performed to compare the rhythmicity of the flies under two temperature conditions. (N = total number of flies, Narr = no. of arrhythmic flies, Nr = no. of rhythmic flies, %r = percentage of rhythmic flies, D. melanogaster Canton-S, I26 × tim19 and I26 × tim21 show more rhythmicity on 25 °C while *D. pseudoobscura* and I26 lines showed preference for 18 °C.

	25 °C						18 °C								
Genotypes	N	Narr	Nr	%r	Period	SEM	N	Narr	Nr	%r	Period	SEM	Chi sq.	Pref	Period
*D. melanogaster* Canton-S	25	1	24	96	23.95	0.13	22	11	11	50.00	23.64	0.08	*	25 °C	
*D. pseudoobscura* Flagstaff	30	20	10	33	23.70	0.16	31	4	27	87.00	22.67	0.05	*	18 °C	*
*w/Y*; *tim*^*01*^*tim*^*mel*^*/tim*^*01*^ (t28s)	23	0	23	100	23.20	0.16	28	6	22	80.00	23.00	0.16			
*per*^*01*^*/Y*, *w; per*^*mel*^/+ (2A)	30	0	30	100	24.87	0.06	11	0	11	100.00	24.01	0.07		25 °C	
*per*^*01*^*/Y*; *tim*^*+*^; *per*^*ps*^*/+* (*I26*)	28	9	19	67.85	31.23	0.46	30	1	29	96.60	26.77	0.07	*	18 °C	*
*w/Y*; *tim*^*01*^, *tim*^*ps*^*/tim*^*01*^ (tim19)	30	14	16	53.3	20.82	0.39	35	9	26	74.20	21.09	0.21		18 °C	
*per*^*01*^*/Y*; *tim*^*01*^, *tim*^*ps*^*/tim*^*01*^; *per*^*ps*^*/+* (*19* × *126*)	46	19	27	58.69	24.51	0.79	32	28	4	12.95	24.89	0.23	*	25 °C	
*w/Y*; *tim*^*01*^, *tim*^*ps*^*/tim*^*01*^ (*tim21*)	34	9	25	73.5	20.09	0.83	55	22	33	63.93	21.10	0.15		18 °C	
*per*^*01*^*/Y*; *tim*^*01*^, *tim*^*ps*^*/tim*^*01*^; *per*^*ps*^*/+* (*21* × *126*)	41	19	22	53.65	26.54	0.63	63	43	20	34.60	24.77	0.15	*	25 °C	
*per*^*01*^*/Y*; *tim*^*01*^, *tim*^*ps*^/*tim*^*01*^; *per*^*ps*^/*+* (*I26* × *35*)	27	21	6	22.22	23.99	0.18	26	17	9	34.60	25.16	0.43			
*w/Y*; *tim*^*01*^, *tim*^*ps*^/*tim*^*01*^ (*tim35*)	32	12	20	61.2	22.32	0.15									
*per*^*01*^*/Y*; *+*; *per*^*ps*^/*per*^*ps*^ (*I26*)	26	6	20	76.92	29.60	0.48	34	4	30	88.20	26.08	0.09			*
*w/Y*; *tim*^*01*^, *tim*^*ps*^*/tim*^*01*^, *tim*^*ps*^ (*19*)	28	17	11	39.2	26.16	0.23	31	19	12	38.70	24.39	0.38		18 °C	
*per*^*01*^/*Y*; *tim*^*ps*^*tim*^*01*^/*tim*^*ps*^*tim*^*01*^; *per*^*ps*^*/per*^*ps*^ (*19* × *126*)	47	46	1		0.00		21	19	2	9.50	22.68	0.17	na		
*w/Y*; *tim*^*01*^, *tim*^*ps*^*/tim*^*01*^ (*21*)	53	30	23	4.35	25.79	0.50	29	17	12	41.30	22.75	0.13		18 °C	
*per*^*01*^/*Y*; *tim*^*ps*^*tim*^*o1*^/*tim*^*ps*^*tim*^*01*^; *per*^*ps*^*/per*^*ps*^ (*21* × *126*)	31	26	5	16.1	21.00	0.72	29	17	12	11.40	22.39	0.12			

All these results described above were obtained with hemizygous single copies of the relevant transgenes. The homozygous *per*^*ps*^ (*I26*) line showed very high levels of rescue at both temperatures but in combination with homozygous *tim*^*ps*^ transgenes, the rescue was extremely poor (Fig. 2, Table 2). Consequently, the results so far suggest that having both heterospecific *per* and *tim* transgenes in a *melanogaster* host compromises the normal and species-specific functioning of the clock and thus do not support any kind of coevolutionary scenario.

**Fig. 3:**
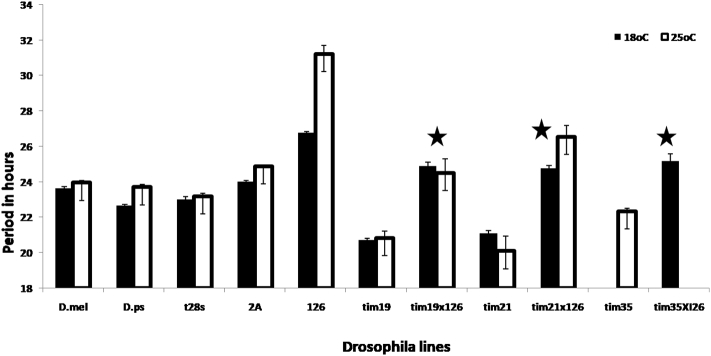
Mean period (±sem) of locomotor activity of the different lines under 18 and 25 °C. The lines indicated with stars are the hemizygous double transgenics (carrying *per* and *tim* transgenes from *pseudoobscura*).

The average period of locomotor activity rhythm for the *per*^*ps*^ transgenic strains *I26* and *I20* fell between 30–32 h whereas *tim*^*ps*^ (lines *tim19*, *tim21* and *tim35*) had shorter mean periods of 20–22 h (Table 2, Fig. 3). The doubly transgenic lines (*tim*^*ps*^*-tim19*, *21* and *35* crossed with *per*^*ps*^ *I26*) (Table 2) showed periods that were very close to 24 h in two of the crosses involving *tim*^*ps*^ *21* and *35*. However, the *tim*^*ps*^ *19/per*^*ps*^ *I26* transformants gave a period of 26 h which is almost exactly intermediate between the values of the two parental lines, as did the corresponding transformants with *tim*^*ps*^*21.* Consequently two of the crosses show results consistent with PER-TIM coevolution whereas the others may simply reflect an averaging of the two parental values.

There is a significant effect of temperature on the period of activity rhythms for *D. pseudoobscura*, as well as for the *D. melanogaster per*^*ps*^ transgenic lines (*I26*) (F_(1,26)_ = 26.7, p = 2.15e−05 and F_(1,35)_ = 68.9, p = 8.79e−10 respectively) (Fig. 3). The two *tim*^*ps*^ transgenic lines tested at both temperatures did not have defective temperature compensation. When *tim*^*ps*^ was combined with *per*^*ps*^ the double transgenic *per*^*01*^/*Y*; *tim*^*01*^, *tim*^*ps*^*/tim*^*01*^; *per*^*ps*^*/+* also showed good temperature compensation (Fig. 3, Table 2). Thus combining the two transgenes from *D*. *pseudoobscura* not only yielded an optimisation in the average period but also produced more optimal temperature compensation.

### 3.3 Locomotor activity profiles under LD 12:12 conditions

**Fig. 4:**
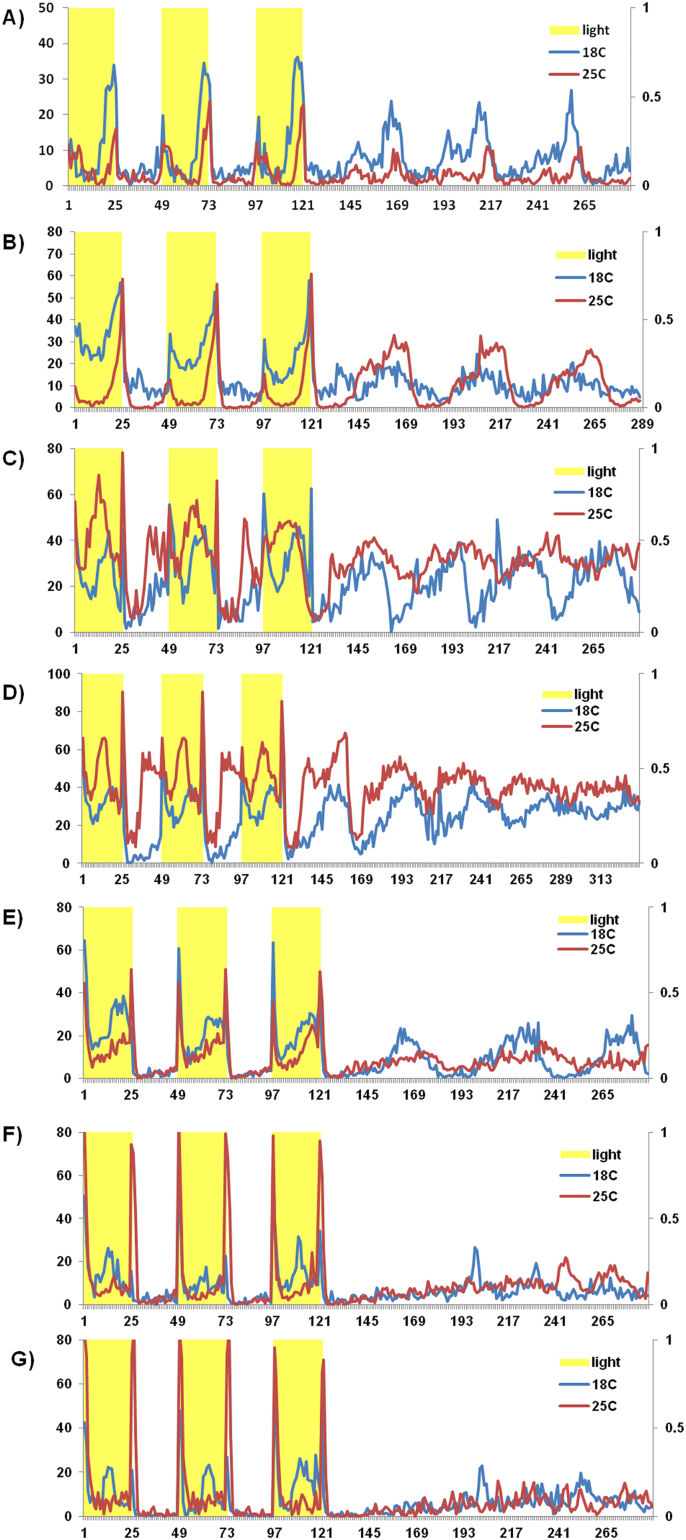
Locomotor activity profile of different fly lines under 18 and 25 °C in three days of LD followed by 3 days in DD (yellow boxes shows photoperiod in LD 12:12). Level of activity (y-axis) plotted against bins of activity (x-axis). A) *D. pseudoobscura* (Flagstaff), B) *D. melanogaster* Canton-S, C) *w*; *tim*^*01*^, *tim*^*ps*^*/tim*^*01*^ (*tim19*), D) *w*; *tim*^*01*^, *tim*^*ps*^*/tim*^*01*^ (*tim21*), E) *per*^*01*^; *tim*^*+*^; *per*^*ps*^*/+* (*I26*), F) *per*^*01*^*/Y*; *tim*^*01*^, *tim*^*ps*^*/tim*^*01*^; *per*^*ps*^*/+* (*19*), G) *per*^*01*^*/Y*; *tim*^*01*^, *tim*^*ps*^*/tim*^*01*^; *per*^*ps*^*/+* (*21*). (For interpretation of the references to colour in this figure legend, the reader is referred to the web version of this article.)

Fig. 4 shows the activity of flies under LD12:12 cycles and for the first few days of DD. *D. pseudoobscura* (Fig. 4A) and *D. melanogaster* both show generally higher levels of activity under 18 °C compared to 25 °C under LD12:12 Interestingly in DD this is reversed in *D. melanogaster* but not *D. pseudoobscura*. The *tim*^*ps*^ transformants (*tim19* and *tim21*, Fig. 4C and D respectively) show considerable nocturnal activity under LD12:12 and, very surprisingly, an ‘afternoon’ peak at 25 °C in addition to the usual morning (M) and evening (E) peaks. In DD the cycles of activity appear to damp quite quickly at the higher temperature. The *per*^*ps*^ *I26* transformant shows higher levels of DD activity at the colder temperature, mimicking *D. pseudoobscura* (compare panel 4E to 4A) and the doubly transgenic flies show a strong afternoon peak at the cold temperature and less so at the warmer one. In DD the damping is immediate (Fig. 4F and G).

### 3.4 Expression studies

**Fig. 5:**
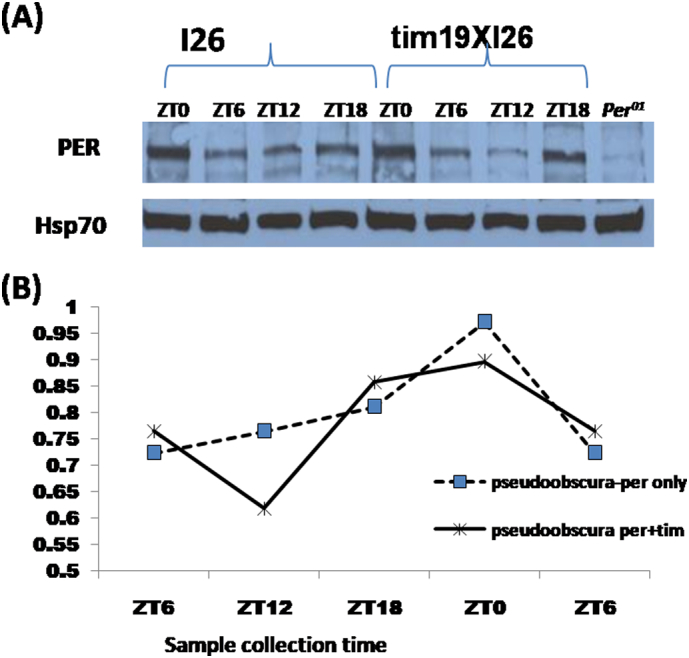
Comparison of PER protein oscillation against HSP70 in *per*^*01*^*/Y*; *tim*^*+*^; *per*^*ps*^*/+* (*I26*) line and *per*^*01*^*/Y*; *tim*^*01*^, *tim*^*ps*^*/tim*^*01*^; *per*^*ps*^*/+* (*19*) lines under LD12:12 A) Western blot indicating PER protein expression (Hsp70 was used as a loading control), B) Graph representing level of protein plotted against the time of sample collection.

We wished to examine the levels of expression of the various transgenes. Expression of PER was compared by using Western blotting. The analysis was performed by collecting the fly heads at 25 °C. Unlike the *D. melanogaster* PER antibodies, the corresponding TIM reagents did not recognise *D. pseudoobscura* TIM on the blots (Fig. 5A). We therefore compared the single *per*^*ps*^ transgenic (*I26*) line and the double transgenic *tim*^*ps*^*19* × *per*^*ps*^ *I26* and *w*; *per*^*01*^ was used as the negative control (Fig. 5). The usual cycling PER pattern with peaks of expression around ZT24/0 were observed even though the double transgenic flies were largely behaviourally arrhythmic in DD (Fig. 5A).

**Fig. 6:**
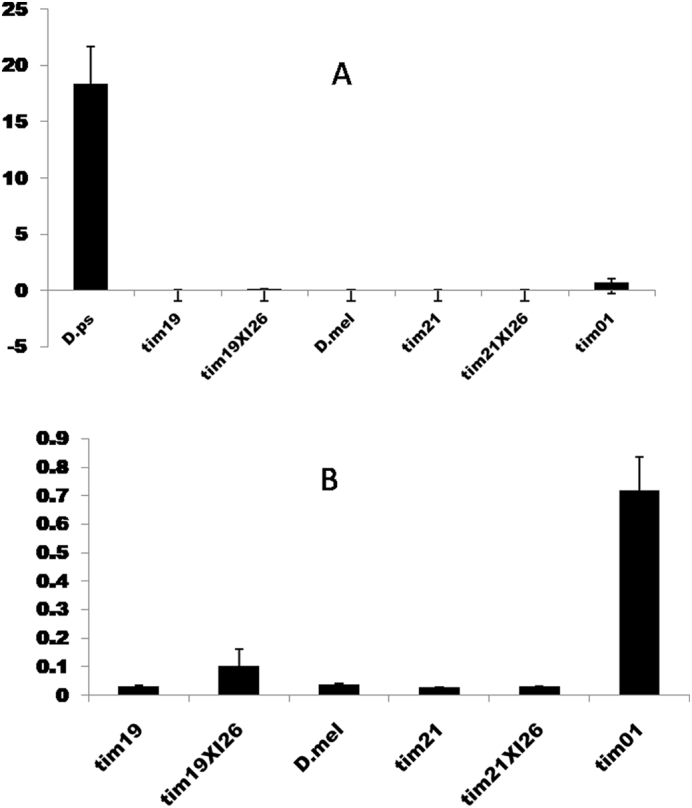
Comparison of mean *tim* mRNA level (±sem) among the different fly lines with and without *D. pseudoobscura*.

As the TIM antibodies were not recognising the *D. pseudoobscura* protein the expression of the *tim*^*ps*^ transgene was investigated with Real-Time PCR on transgenic *tim*^*ps*^ *19* and *tim*^*ps*^ *21*, the double transgenic *tim*^*ps*^*19* × *per*^*ps*^ *I26* and *tim*^*ps*^*21* × *per*^*ps*^ *I26* and *D. pseudoobscura* and *D. melanogaster*. Fly heads were collected at ZT12 from individuals maintained at 25 °C. Three samples of each fly line were collected at ZT12 and three technical replicates were used for each. The fly line *w*; *tim*^*01*^ was used as the negative control. The analysis showed that the level of *tim* in *D. pseudoobscura* is far higher than all the other lines as was *tim*^*01*^, which is expected as the negative auto-regulation by TIM is removed in the mutant (Fig. 6A). A statistical analysis was performed by excluding *D. pseudoobscura*. Results revealed that only *tim*^*mel*^ (t28s) has a significantly higher level of *tim* mRNA compared to all other transgenes(F_(5,47)_ = 3.51, p = .009, Fig. 6B). However the very low relative levels of *tim* transcript are clearly sufficient to drive circadian rhythmicity, both in the wild-type and transgenics.

## 4 Discussion

### 4.1 Protein sequence alignment

*D. pseudoobscura* separated from the *D*. *melanogaster* group between 25 (Russo et al., 1995) and 30 Mya (Schlötterer et al., 1994). However for both PER and TIM, the position of the *D. pseudoobscura-persimilis* clade is further away than is *D. virilis,* which itself diverged from *D. melanogaster* 40–60 Mya (Powell and DeSalle, 1995) (Fig. 1A, B). The position of *D. willistoni* is also not in accordance with the reference sequence. This may suggest some kind of non-neutral evolution of these proteins, and provides an additional rational for further exploring the coevolutionary idea.

For *D. pseudoobscura* the highest level of similarity was obtained as expected with its sibling North American species *D. persimilis*. Two PER interaction domains have been identified on TIM, PER1 and PER2 (Saez and Young, 1996). Piccin et al. (2000) in their phylogeny, reported the canonical phylogenetic positions when using the full PER protein sequence but by comparing only the PAS interaction domains of PER, *M. domestica* was grouped closer to *D. melanogaster* than *D. pseudoobscura*. This corresponded with the enhanced rescue of *Musca* PER compared to *D. pseudoobscura* PER in *D. melanogaster per*^*0*^ hosts. The same was observed with the comparison of the PAS region of *Ceratitis capitata* PER (Mazzotta et al., 2005). PAS is the protein-protein interaction domain important for signalling and sensory function. In *Drosophila* its function is to promote the interactions of PER with TIM, and CLK with CYC (Mazzotta et al., 2005). However on PER there is a CLOCK-CYCLE Domain (CCID) for interaction with CLK (Chang and Reppert, 2003).

The unusual phylogenetic position of PAS may be due to the amino acids in this region being under selective constraints so it does not evolve independently. It may coevolve in concert with the dimerization domains of its conspecific molecular partners (Mazzotta et al., 2005). Perhaps the PER interaction domain of TIM should also reflect the unusual phylogeny of the PAS domain of its partner PER. However in the present study the results of the phylogenetic analysis using the amino acid sequence of only PER1 and PER2 interacting domains of TIM gave the same position for *D. pseudoobscura* as that of the full TIM sequence.

### 4.2 Coevolution

Coevolution is an evolutionary process in which a heritable change in one entity establishes selective pressure for a change in another entity. These entities can range from nucleotides to amino acids to protein to entire organism and perhaps the whole ecosystem. The well-studied example of coevolution involves physically interacting proteins in which precise, complementary structural conformations of interacting partners are needed to maintain a functional interaction (Fraser et al., 2004). The basic aim of the current study was to investigate whether PER and TIM form a coevolved module that can interact more efficiently with other clock proteins compared to the situation where only one of these negative regulators was heterospecific. For this purpose transgenes of *per* and *tim* from *D. pseudoobscura* were introduced into *D. melanogaster* host.

The comparison of the level of rescue in the rhythmic behaviour of hemizygous/homozygous transgenics with single *per* or *tim* or both was performed. Previous studies using *per*^*ps*^ transformants showed that *per*^*ps*^ cannot rescue rhythmic behaviour efficiently. It has low penetrance (30–50%) and flies have very long average periods (Petersen et al., 1988; Peixoto et al., 1998). The same observations were confirmed in the present study. The level of rescue for the *tim*^*ps*^ transgenic flies was also <50% under 25 °C but increased at 18 °C. The period of these lines was short (21–22 h). These results suggested that the two *pseudoobscura* proteins are unable to interact fully with their *melanogaster* partner molecules. The rescue observed in *tim*^*01*^ flies using *D. virilis* (Ousley et al., 1998) and *D. ananassae tim* transgenes (Nishinokubi et al., 2006) corresponds to the position of these flies with respect to *D. melanogaster* in the phylogenetic analysis.

When both heterospecific transgenes were studied simultaneously in double mutant hosts, no significant increase in levels of rhythmicity was obtained, indeed it was reduced suggesting that adding additional heterospecific clock proteins or even increasing the dose of one interspecific molecule, was disruptive for the host clock – the opposite of coevolution. However a significant and dramatic improvement in the average period of the hemizygous double transgenics was observed. The average period ranged from 24 to 26.5 h for all three lines (tim*19*, *tim21* and *tim35*), While this might initially appear to fit with a coevolution hypothesis, it was also noticeable how some of the double transgenic periods appeared intermediate to the parental single transgenic values. Consequently, we cannot securely state that these results are reflecting a more optimal PER-TIM interaction of the heterospecific clock proteins.

The activity profiles of the transgenic flies showed some aspects of the species-specific behaviour controlled by the clock genes. For example, the *per*^*ps*^ *I26* line showed higher levels of activity in the DD phase at colder temperatures, similar to *D. pseudoobscura*. The most interesting novel phenotype was the afternoon peak of locomotor activity in LD12:12 observed in the single *8b* transformants. This is reminiscent of the same afternoon component that is observed in wild-type when exposed to naturally cycling warm summer temperatures but is not generally seen under laboratory square wave lighting conditions at constant temperature (Vanin et al., 2012; Green et al., 2015). However, given the generally shorter endogenous period of the transformants, the apparent afternoon peak may represent an earlier phased E peak that is followed by a startle response when the lights go off, which would superficially resemble an E peak.

The bimodal activity profile of *D. melanogaster* is under the control of several sets of neurons (Helfrich-Förster, 2005). Under LD conditions in the laboratory, the PDF-positive sLNvs have been implicated in control of the morning locomotor activity forming a neuronal basis for the morning (M) oscillator, while the fifth PDF-negative sLNv and LNds seem to be responsible for the evening activity and hence termed the evening (E) oscillator (Grima et al., 2004; Nitabach and Taghert, 2008; Stoleru et al., 2004). The activity profile of *D. pseudoobscura* did not show any morning anticipation of activity in LD. This may be an adaptive response of this species where the morning activity lost its selective advantage due to the long summer days of more northern climatic zones (Hermann et al., 2013). This type of activity profile has also been reported from high latitude species *D. montana* (Kauranen et al., 2012), *D. virilis* (Dubruille and Emery, 2008), *D. ezoana* and *D. littoralis* (Menegazzi et al., 2017). These species do not express PDF in the sLNv's (or express at very low levels), (Kauranen et al., 2012) and the latter two species do not express CRY in the lLNvs (Menegazzi et al., 2017). Hermann et al., 2013 reported some apparent differences in the PER expression in the clock neurons between *Sophophora* (to which *D. pseudoobscura* belongs) and *Drosophila* subgenuses. Indeed, they reported that *D. pseudoobscura* also showed reduced PDF immunostaining in the sLNvs which might be expected to generate weaker rhythms, given the prominent role of these neurons as the pacemaker cells in DD (Nitabach and Taghert, 2008).

### 4.3 Effect of temperature

The analysis of locomotor activity of the different fly strains under two different temperature conditions gave some interesting results. In *D. melanogaster* the locomotor peak of activity is temperature modulated so that with a rise in temperature the E (evening) peak of activity moves later in the day, generating a mid-day siesta allowing the fly to avoid the desiccating effect of the hottest part of the day (Majercak et al., 1999; Collins et al., 2004; Low et al., 2008). *D. pseudoobscura* clearly favoured colder temperatures for expressing rhythmic behaviour while *D. melanogaster* flies were more rhythmic under higher temperature conditions. Hennessy (1999) also reported similar observation with *D. melanogaster* transgenes and *per*^*ps*^ transgenes favouring warmer and colder conditions respectively. In the current study, the *tim*^*mel*^ (*t28s*) behaves like Canton-S but the *tim*^*ps*^ transgenic line *tim19* favoured colder conditions. This suggested that in restoring wild-type clock function, the *tim*^*ps*^ transgene takes on a dominant effect. Such dominant effects of *pseudoobscura* transgenes in mating rhythms have been seen before with *per*^*ps*^ in *per*^*+*^ backgrounds (Petersen et al., 1988; Tauber et al., 2003). These observations reveal that restoring TIM from *D. pseudoobscura* in *tim-null* mutants appears to generate *pseudoobscura* like colder temperature characteristics.

An overall trend toward shortening of period under 18 °C was seen in the controls (both natural and transgenic) and *per*^*ps*^ transformant lines. The same trend was reported in previous studies using these fly lines (Piccin et al., 2000; Hennessy, 1999; Peixoto et al., 1998). However the difference in the average period for the *per*^*ps*^ flies was large and >4 h. Compared to the *per*^*ps*^ transgenics, the temperature compensation was much better in single *tim*^*ps*^ and double *per*^*ps*^ and *tim*^*ps*^ transgenics. These results suggested that temperature compensation is disturbed more by heterospecific PER molecules than TIM molecules. This would fit with the general view that PER is more important for thermal adaptation of the clock (Majercak et al., 2004; Sawyer, 1997) than TIM, the latter being more relevant for light sensitivity of the clock (Zeng et al., 1996) and associated photoperiodic phenotypes such as diapause (Tauber et al., 2007).

The phase analysis of the locomotor activity in the transgenic flies in LD12:12 also showed some very interesting results. Bywalez et al. (2012) found that M and E components of locomotor activity do not occur at a fixed time and respond differently to day length and temperature. They suggested that the two underlying oscillators have different sensitivities and the phase of evening activity is more sensitive to high temperature, resulting in a delay. The phase of the morning peak of activity was compared for the transgenic lines and it was revealed that *tim*^*ps*^ transgenics are active earlier under 25 °C than 18 °C which is the normal heat avoiding response by *D. melanogaster* flies, controlled at least partially through the reduced 3′ splicing of the *per* transcript (Majercak et al., 1999; Low et al., 2008). This splicing also delays the E peak in hot days, generating the siesta. However the single *tim*^*ps*^ transformants flies cannot adjust their evening peak according to the temperature conditions and showed an earlier E peak on hotter rather than colder temperatures. Combining *tim*^*ps*^ with *per*^*ps*^, produced a later evening peak at 25 °C than at 18 °C so the normal hot day response requires both heterospecific PER and TIM partners, revealing a possible example of coevolution.

In summary, our results provide limited support for a coevolution of PER and TIM within species, and certainly not for maintaining robust levels of rhythmicity. Some positive evidence was found for the free-running period of activity rhythm and there were some other sporadic examples where having PER and TIM from the same species gave a more optimal circadian phenotype in our transgenics. There were also some examples of apparent species-specific behaviour associated with the corresponding transgenes. However in the main, we have not showed any compelling evidence for PER-TIM representing a co-evolved module as initially hinted at by the phylogenetic and functional analyses of PER by Piccin et al. (2000).

The following is the supplementary data related to this article.

Supplementary Fig. 1 Genetic crosses showing A) the lines carrying *D*. *pseudoobscura-tim* on chromosome II were crossed to the double balancers (P) to combine the transgenes with markers on chromosome III. B) The lines carrying *D*. *pseudoobscura-per* on chromosome III were crossed to the double balancers (P) to combine the transgenes with markers on chromosome II. C) The final strains obtained from cross A and B were crossed with each other to combine the two transgenes of *D*. *pseudoobscura per* and *tim* in the double mutant background with only male flies. D) Final cross to obtain the homozygous transformant female flies having two copies of *D*. *pseudoobscura-tim* and *per* in the *per*^*0*^; *tim*^*0*^ background. The triple balancer *FM7a/per*^*01*^; *CyO/Sco*; *MKRS/+* females were crossed to the *w/Y*; *tim*^*01*^, *tim*^*ps*^*/tim*^*01*^, *tim*^*ps*^; *per*^*ps*^*/per*^*ps*^ males. Supplementary Fig. 1
