# Supplementary material for: Interspecific studies of circadian genes period and timeless in Drosophila
Source: Gene. 2018 Mar 30;648:106–14. doi: 10.1016/j.gene.2018.01.020 (PMC5818170; doi:10.1016/j.gene.2018.01.020)
Supplement: Supplementary Fig. 1 — Genetic crosses showing A) the lines carrying D. pseudoobscura-tim on chromosome II were crossed to the double balancers (P) to combine the transgenes with markers on chromosome III. B) The lines carrying D. pseudoobscura-per on chromosome III were crossed to the double balancers (P) to combine the transgenes with markers on chromosome II. C) The final strains obtained from cross A and B were crossed with each other to combine the two transgenes of D. pseudoobscura per and tim in the double mutant background with only male flies. D) Final cross to obtain the homozygous transformant female flies having two copies of D. pseudoobscura-tim and per in the per0; tim0 background. The triple balancer FM7a/per01; CyO/Sco; MKRS/+ females were crossed to the w/Y; tim01, timps/tim01, timps; perps/perps males. [file mmc1.pdf]

P ♀ ***w/w; tim^{01}, tim ^{ps}/ tim^{01}, tim ^{ps};+/+*** **X** ♂ ***w/Y; CyO/Sco; TM6b/MKRS***

(Orange eye *pseudoobscura –tim* transgenics) **(*white* eye double balancers)**

F1 ♀ ***w/w; tim^{01},tim ^{ps}/CyO; TM6B/+*** **X** ♂ ***+/Y; tim^{01},tim ^{ps}/CyO; MKRS/+***

(Collect virgins which are both *CyO* and *TM6B*) **(Collect males which are both *CyO* and *MKRS*)**

F2

w/w/Y; tim^{01},tim ^{ps} / tim^{01} , tim ^{ps} ; TM6b/MKRS
and
w/w/Y; tim^{010},tim ^{ps} / CyO ; TM6b/MKRS

P ♂ ***per^{01}* w+/ Y*; +; per ^{ps}/per ^{ps}*** **X** ♀ ***w/w; CyO/Sco; TM6b/MKRS***

(*rosy^{+}* eyed *pseudoobscura-per* transgenics) **(*white* eye double balancers)**

F1 ♀ ***per^{01},w+/w; CyO/+; per ^{ps}/TM6B*** **X** ♂ ***w/Y; Sco/+; per^{ps}/TM6B***

F2

w; Cyo/Sco; per ^{ps}/ per ^{ps}
and
w; Cyo/Sco; per ^{ps}/TM6B

P ♀ ***w/w, tim^{01}, tim^{ps}/; tim^{01}, tim^{ps}; TM6b/MKRS*** **X** ♂ ***w/Y; Cyo/Sco; per ^{ps}/ per ^{ps}***

(F2 flies from 1st cross) **(F2 flies from 2nd cross)**

F1 ♀ ***w/w; tim^{01},tim ^{ps}/ CyO; per ^{ps}/ TM6B*** **X** ♂ ***w/Y; tim^{01},tim ^{ps}/ CyO; per ^{ps}/***

F2 ♂ ***per/Y $^{+}$; tim^{01},tim ^{ps}/ tim^{01},tim ^{ps}; per ^{ps}/ per ^{ps}*** ♀ ***per^{01} w, / per^{01} w; tim^{01}/tim^{01}; +/+***

(Homozygous flies containing the both transgenes but *per^{+}* background)

per^{01} w/Y ; tim^{01},tim ^{ps}/ tim^{01}; per ^{ps}$_{/+}$
(Hemizygous double transgenic males in the double mutant background)

P

♀ $FM7a/per^{01}; CyO/Sco; MKRS/+$
(Female with balancers on three chromosomes with per^{01} **on X marked with w)**

X

♂ $w/Y; tim^{01}, tim^{ps}/ tim^{01}, tim^{ps}; per^{ps}/ per^{ps}$
(Homozygous flies containing the both transgenes but per^{+} **background)**

F1

♀ $per^{01}/w; CyO/ tim^{ps}; per^{ps}/MKRS$
(Backcrossed to P1 males)

F2

♂ $per^{01}/Y ; tim^{01}, tim^{ps}/ tim^{01}, tim^{ps}; per^{ps}/ per^{ps}$
(Select the male flies without markers and perform wing PCR. Then backcross the per^{01} **males with F1 females)**

$per^{01}/Y; tim^{01}, tim^{ps}/ tim^{01}, tim^{ps}; per^{ps}/ per^{ps}$
(Collect virgin flies, cut wings, genotype them and cross the homozygous transformants in the double mutant background)
